# Supplementary material for: Nelfb promotes dermal white adipose tissue formation through RNA polymerase II-mediated adipogenic gene regulation
Source: Development. 2025 Oct 10;152(20):dev204976. doi: 10.1242/dev.204976 (PMC12539206; doi:10.1242/dev.204976)
Supplement: Supplementary information [file develop-152-204976-s1.pdf]

Supplemental Figure 1

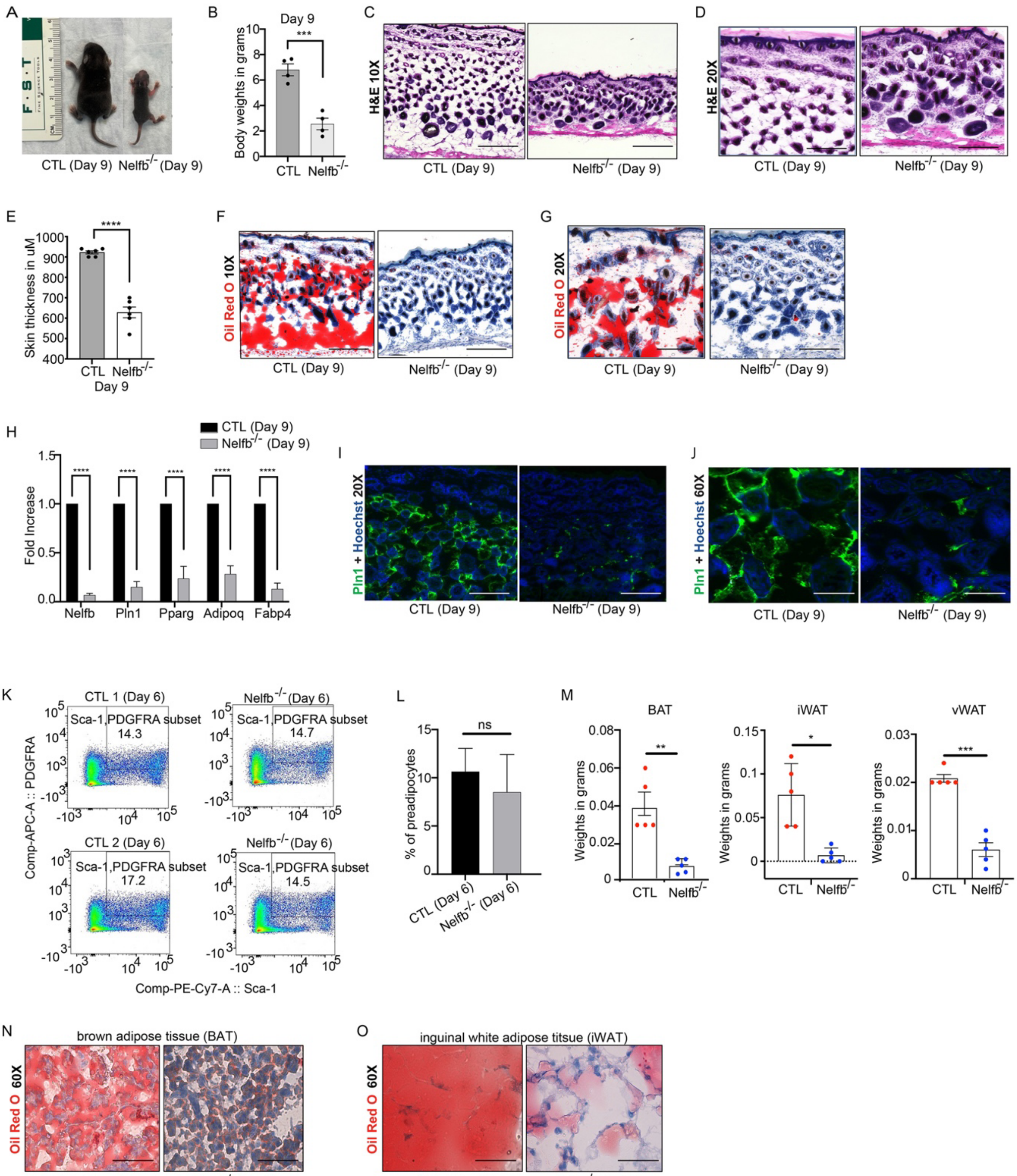

Fig. S1. Nelfb is essential for the formation of white and brown adipose tissue. **(A)** Images of control (CTL: *Nelfb*$^{fl/wt}$ *Pdgfra-Cre*+ or *Nelfb*$^{wt/wt}$ *Pdgfra-Cre*+) and *Nelfb*$^{-/-}$ (Nelfb$^{fl/fl}$ Pdgfra-Cre+) mice at postnatal day 9. **(B)** Body weights of CTL and *Nelfb*$^{-/-}$ mice at day 9. All individual dots in bar graphs represent data from an individual mouse (N=4). **(C-D)** Hematoxylin and Eosin staining of CTL and *Nelfb*$^{-/-}$ dorsal skin harvested at day 9 at 10X (C) and 20X (D) magnification (N=4). **(E)** Quantification of CTL and *Nelfb*$^{-/-}$ dorsal skin thickness from animals harvested at day 9 (N=6). **(F-G)** Oil Red O staining of CTL and *Nelfb*$^{-/-}$ dorsal skin harvested at day 9 at 10X (F) and 20X (G) magnification (N=4). **(H)** RT-qPCR of adipogenic genes on CTL and *Nelfb*$^{-/-}$ dermis at day 9 (N=4). Results were normalized to house-keeping gene, *Gapdh*. Mean values are shown with error bars representing SEM. ****p<0.0001 (two-tailed unpaired t tests). **(I-J)** Staining of dorsal skin from CTL and *Nelfb*$^{-/-}$

Development • Supplementary information

mice with antibodies against Pln1 (Pln1; mature adipocyte marker: green) and Hoechst 33342 (nuclei stain: blue) at 20X (I) and 60X (J) magnification (N=3). **(K)** Representative flow cytometry plots for gating preadipocytes. The plot shows Pdgfra/Sca-1 double positive cells gated from dorsal dermis of CTL and *Nelfb*$^{-/-}$ mice at postnatal day 6. Cells were first gated based on FSC and SSC, followed by exclusion of dead cells. This is followed by gating for CD45-/CD31- population to remove immune and endothelial cells. Pdgfra/Sca-1 was gated from CD45-/CD31- population. **(L)** Quantification of preadipocytes from dorsal dermis of CTL and *Nelfb*$^{-/-}$ mice (N=4). Mean values are shown with error bars representing SEM, (Two tailed paired t tests). NS=not significant. **(M)** Weight of interscapular brown adipose tissue (BAT), inguinal white adipose tissue (iWAT), and mesenteric visceral white adipose tissue (vWAT) from CTL and *Nelfb*$^{-/-}$ mice at postnatal day 6. Each dot represents weight of tissue from an individual animal. **(N)** Oil Red O staining of CTL and *Nelfb*$^{-/-}$ brown adipose tissue harvested at day 6 (N=4). **(O)** Oil Red O staining of CTL and *Nelfb*$^{-/-}$ inguinal white adipose tissue harvested at day 6 (N=2). Scale bar=50μm for 60X, 150μm for 20X, 300μm for 10X.

Development • Supplementary information

Supplemental Figure 2

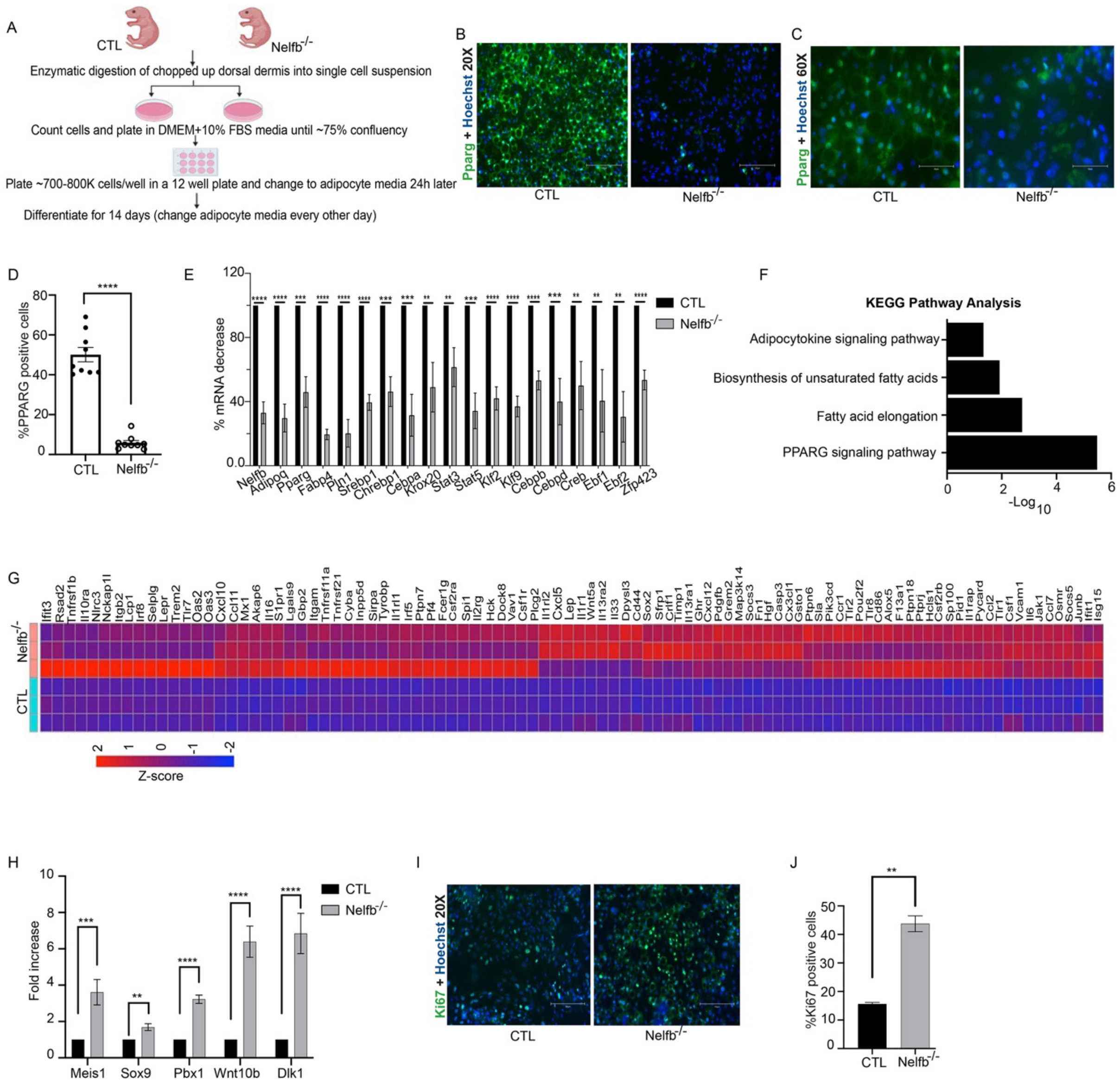

Fig. S2. Nelfb promotes adipocyte differentiation.

(A) Schematic diagram of cell isolation from postnatal day 0 dermis harvested from control (CTL: *Nelfb*$^{fl/wt}$ *Pdgfra-Cre*+ or *Nelfb*$^{wt/wt}$ *Pdgfra-Cre*+) and *Nelfb*$^{-/-}$ (Nelfb$^{fl/fl}$ Pdgfra-Cre+) mice. Isolated cells were cultured in adipocyte differentiation medium for 14 days. **(B-C)** Staining of CTL and *Nelfb*$^{-/-}$ cells with antibodies against Pparg (green) and Hoechst 33342 (nuclei stain: blue) at 20X (B) and 60X (C) magnification (N=4). **(D)** Percent of Pparg+ cells were quantified from 60X images (N=3). **(E)** RT-qPCR of adipogenic genes on CTL and *Nelfb*$^{-/-}$ cells (N=3). Results were normalized to house-keeping gene, *Gapdh*. **(F)** KEGG Pathway analysis of 358 downregulated genes in *Nelfb*$^{-/-}$ cells using Enrichr. **(G)** Heat map of the top 100 upregulated genes (≥2-fold change) in *Nelfb*$^{-/-}$ cells compared to CTL adipocytes. **(H)** RT-qPCR of inhibitors of adipogenesis (preadipocyte genes) on CTL and *Nelfb*$^{-/-}$ cells cultured in adipocyte differentiation medium for 14 days (N=4). **(I)** Staining of CTL and *Nelfb*$^{-/-}$ cells with antibodies against Ki67 (green) and Hoechst 33342 (nuclei stain: blue) (N=4). **(J)** Percent of Ki67+ cells were quantified from 60X pictures. Mean values are shown with error bars representing SEM. **p<0.01, ***p<0.001, ****p<0.0001 (two-tailed unpaired t tests). Scale bar=50μm for 60X, 150μm for 20X.

Development • Supplementary information

Supplemental Figure 3

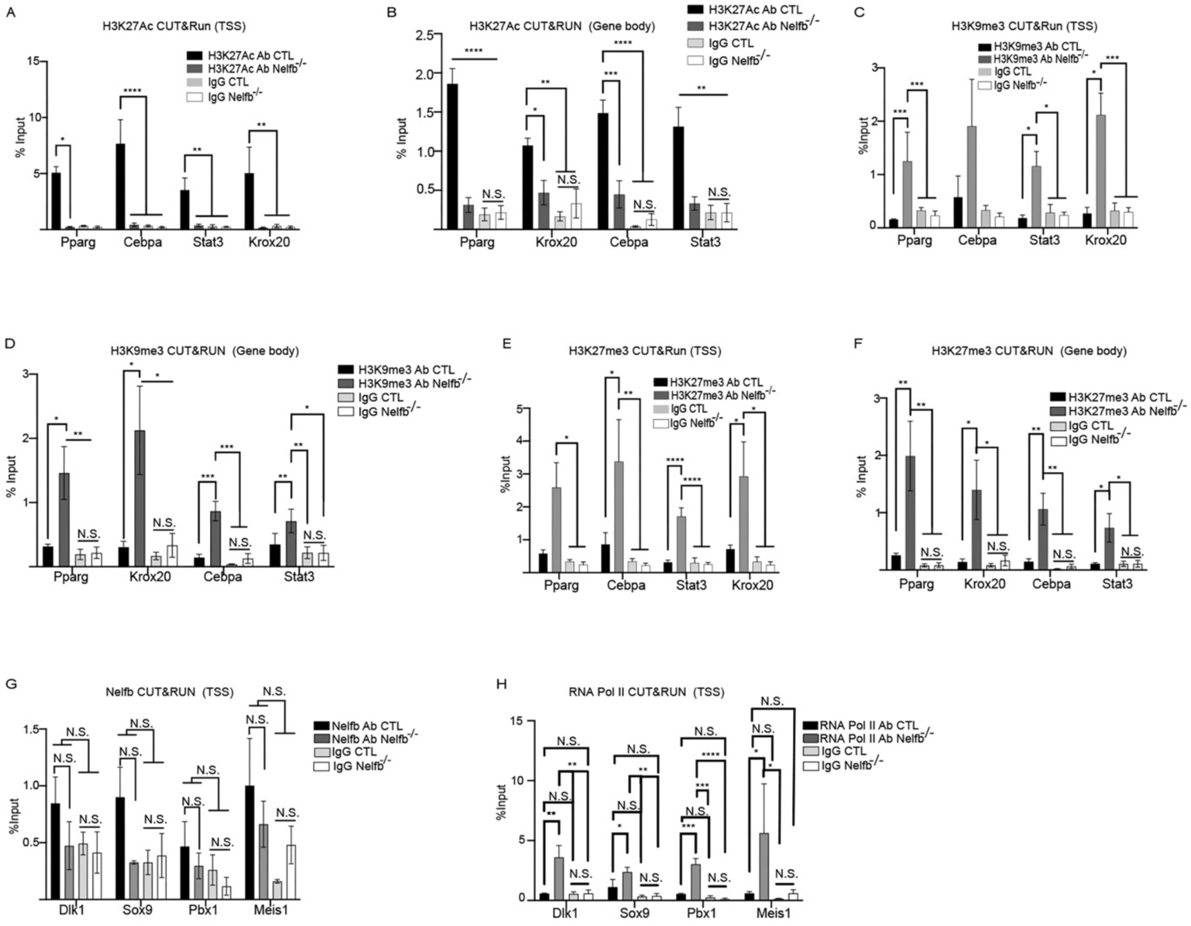

Fig. S3. Nelfb is required for RNA Polymerase II stabilization and maintaining open chromatin at genes coding for adipocyte differentiation promoting transcription factors.

(A-B) CUT&RUN on control (CTL: *Nelfb*$^{fl/wt}$ *Pdgfra-Cre*+ or *Nelfb*$^{wt/wt}$ *Pdgfra-Cre*+) and *Nelfb*$^{-/-}$ (Nelfb$^{fl/fl}$ Pdgfra-Cre+) cells cultured in adipocyte differentiation medium for 14 days using an anti-H3K27ac (open chromatin) antibody and anti-IgG antibody. qPCR analysis was conducted on the TSS (A) and gene body (B) regions of *Pparg, Cebpa, Stat3,* and *Krox20.* Signal was calculated as a percent of total input DNA. **(C-D)** CUT&RUN was performed on CTL and *Nelfb*$^{-/-}$ cells using an anti-H3K9me3 (repressive chromatin) antibody and anti-IgG antibody. qPCR analysis was conducted on the TSS (C) and gene body (D) regions. **(E-F)** CUT&RUN was performed on CTL and *Nelfb*$^{-/-}$ cells using an anti-H3K27me3 (repressive chromatin) antibody and anti-IgG antibody. qPCR analysis was conducted on the TSS (E) and gene body (F) regions. **(G-H)** CUT&RUN on CTL and *Nelfb*$^{-/-}$ cells cultured in adipocyte differentiation medium for 14 days using an anti-Nelfb (G) antibody and an anti-RNA Pol II (H) antibody. qPCR analysis was conducted on transcription start site (TSS) regions of *Dlk1, Sox9, Pbx1,* and *Meis1.* Signal was calculated as a percent of total input DNA. Mean values are shown with error bars representing SEM. N=3 biological replicates for entire figure.

*p<0.05, **p<0.01, ***p<0.001, ****p<0.0001 (one-way ANOVA followed by Tukey's multiple comparison tests). NS=not significant.

Development • Supplementary information

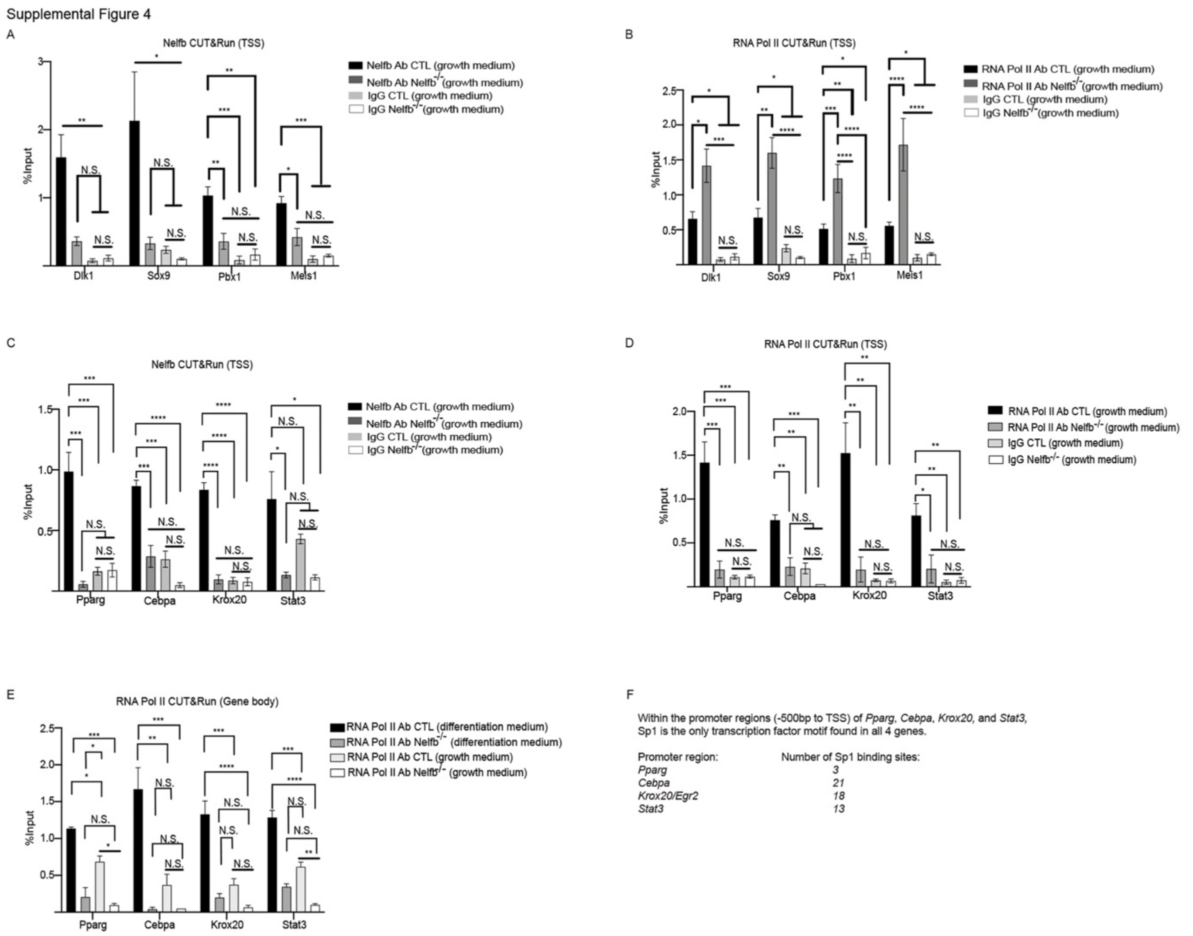

Fig. S4. Nelfb binds and is necessary for RNA Polymerase II stabilization of differentiation transcription factors in adipocyte precursor cells.

(A-B) CUT&RUN was performed on control (CTL: *Nelfb*$^{fl/wt}$ *Pdgfra-Cre*+ or *Nelfb*$^{wt/wt}$ *Pdgfra-Cre*+) and *Nelfb*$^{-/-}$ (Nelfb$^{fl/fl}$ Pdgfra-Cre+) cells using an anti-Nelfb (A) and anti-RNA Polymerase II (B) antibody. qPCR analysis using primers targeting transcription start site (TSS) regions of *Dlk1, Sox9, Pbx1,* and *Meis1.* Cells were cultured in growth medium. **(C-D)** CUT&RUN was performed on CTL and *Nelfb*$^{-/-}$ cells using an anti-Nelfb (C) and anti-RNA Pol II (D) antibody. qPCR analysis using primers targeting TSS regions of *Pparg, Cebpa, Krox20,* and *Stat3.* Cells were cultured in growth medium. **(E)** CUT&RUN was performed on CTL and *Nelfb*$^{-/-}$ cells using an anti-RNA Pol II and anti-IgG antibody. Cells were cultured in growth or adipocyte differentiation medium for 14 days. qPCR analysis using primers targeting gene body regions of *Pparg, Cebpa, Krox20,* and *Stat3.* Signal was calculated as a percent of total input DNA. Mean values are shown with error bars representing SEM. N=3 biological replicates for entire figure. *p<0.05, **p<0.01, ***p<0.001, ****p<0.0001 (one-way ANOVA followed by Tukey's multiple comparison tests). NS=not significant. **(F)** The promoter regions of *Pparg, Cebpa, Krox20, and Stat3* were scanned for transcription factor binding motifs using TRANSFAC. The promoter region was defined as -500bp to the transcription start site of each gene. Sp1 was the only transcription factor binding motif found in all 4 genes.

Development • Supplementary information

Supplemental Figure 5

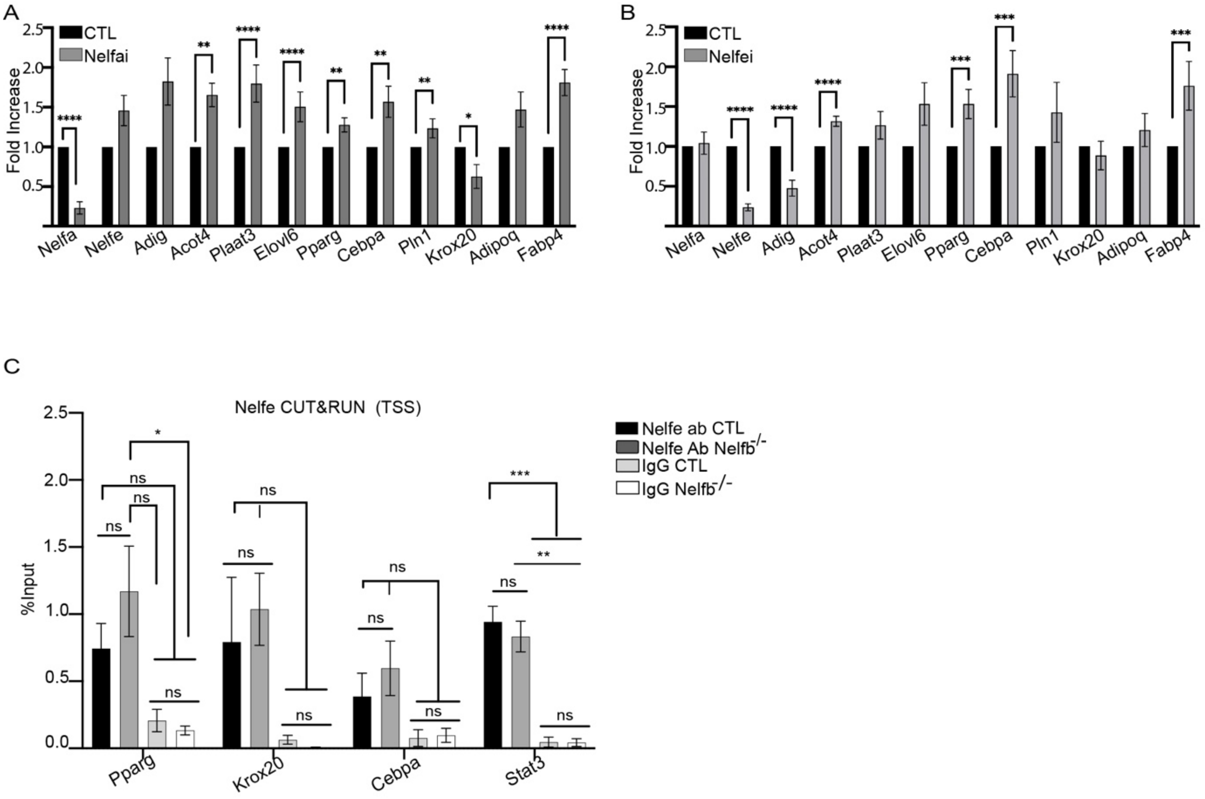

Fig. S5. Nelfa and Nelfe are not necessary for adipocyte differentiation.
(A) Fibroblasts from newborn (P0) wildtype mice were isolated and knocked down using non-targeting siRNAs (Control:CTL) or Nelfa siRNAs (Nelfai). Cells were placed in adipocyte differentiation medium for 7 days and harvested for RNA. RT-qPCR of adipogenic genes was performed. Results were normalized to house-keeping gene, *Gapdh*. **(B)** Fibroblasts were knocked down using CTL or Nelfe siRNAs (Nelfei) and induced to differentiate in adipocyte medium for 7 days. RT-qPCR of adipogenic genes was performed. Results were normalized to house-keeping gene, *Gapdh*. **(C)** CUT&RUN was performed on CTL and *Nelfb*$^{-/-}$ cells using an anti-Nelfe and anti-IgG antibody. Cells were cultured in adipocyte differentiation medium for 14 days. qPCR analysis using primers targeting TSS regions of *Pparg, Cebpa, Krox20,* and *Stat3.* Signal was calculated as a percent of total input DNA. Mean values are shown with error bars representing SEM. *p<0.05, **p<0.01, ***p<0.001, ****p<0.0001 (multiple unpaired t tests for A-B and one-way ANOVA followed by Tukey’s multiple comparison tests for C). N=5 for A-B and N=3 for C.

Table S1. RNA-seq analysis of genes differentially expressed upon deletion of Nelfb in cells cultured in adipocyte differentiation medium. Significantly upregulated or downregulated genes were identified based on FDR ≤ 0.05 and ≥ 2-fold change.

Available for download at
https://journals.biologists.com/dev/article-lookup/doi/10.1242/dev.204976#supplementary-data

Table S2. List of primers and siRNA sequences used. This table provides the sequences of RT-QPCR primers, CUT&RUN primers, genotyping primers, and siRNA sequences.

Available for download at
https://journals.biologists.com/dev/article-lookup/doi/10.1242/dev.204976#supplementary-data

Development • Supplementary information
